# Altered gut microbiome and autism like behavior are associated with parental high salt diet in male mice

**DOI:** 10.1038/s41598-021-87678-x

**Published:** 2021-04-16

**Authors:** Kazi Farhana Afroz, Noah Reyes, Kobe Young, Kajal Parikh, Varsha Misra, Karina Alviña

**Affiliations:** 1 Department of Biological Sciences, Texas Tech University, Lubbock, TX 79409 USA; 2 Honors College, Texas Tech University, Lubbock, TX 79409 USA; 3 Department of Neuroscience, University of Florida, 1395 Center Drive, Room D5-33D, Gainesville, FL 32610-0244 USA

**Keywords:** Neuroscience, Psychology, Diseases, Medical research, Risk factors

## Abstract

Neurodevelopmental disorders are conditions caused by the abnormal development of the central nervous system. Autism spectrum disorder (ASD) is currently the most common form of such disorders, affecting 1% of the population worldwide. Despite its prevalence, the mechanisms underlying ASD are not fully known. Recent studies have suggested that the maternal gut microbiome can have profound effects on neurodevelopment. Considering that the gut microbial composition is modulated by diet, we tested the hypothesis that ASD-like behavior could be linked to maternal diet and its associated gut dysbiosis. Therefore, we used a mouse model of parental high salt diet (HSD), and specifically evaluated social and exploratory behaviors in their control-fed offspring. Using 16S genome sequencing of fecal samples, we first show that (1) as expected, HSD changed the maternal gut microbiome, and (2) this altered gut microbiome was shared with the offspring. More importantly, behavioral analysis of the offspring showed hyperactivity, increased repetitive behaviors, and impaired sociability in adult male mice from HSD-fed parents. Taken together, our data suggests that parental HSD consumption is strongly associated with offspring ASD-like behavioral abnormalities via changes in gut microbiome.

## Introduction

According to statistics of the World Health Organization, approximately 1% of the world population is currently diagnosed with ASD, the most common neurodevelopmental disorder^1,2^. Yet, its etiology is not fully understood. The current consensus is that ASD is likely caused by the combination of genetic, environmental, and neurodevelopmental factors^3^.

Recent studies have shown that the development of CNS is crucially dependent on the infant’s gut microbiome (i.e. microbial composition of the gut flora)^4,5^, which comes primarily from a combination of vaginal or skin microbiota depending on birth type (i.e. vaginal or cesarean)^6,7^, and from the maternal gut microbiota that provides essential metabolites needed for fetal development of both the immune and nervous systems^7^. Seminal studies using germ-free mice revealed abnormal behaviors associated with altered gut microbiome. Such abnormalities include increased locomotor activity, reduced anxiety-like behaviors in the elevated plus maze and changes in signaling pathways involved in synaptic function^8,9^. Additionally, the gut microbiome has been associated with altered gene expression in excitatory neurons of the medial prefrontal cortex as well as deficit in fear extinction learning^10^. Collectively these studies suggest that the gut microbiome can influence the development of the brain and, ultimately, behavior^1,10^. Further, these findings also highlight the fact that the maternal microbiome can have an important role in shaping neurodevelopment in the offspring^11^. In support of this hypothesis, studies using the mouse model of Maternal Immune Activation (MIA, an artificial induction of inflammation by injecting bacterial endotoxin or synthetic virus in the pregnant rodent) showed that maternal probiotic treatment containing specific bacterial strains prevented ASD-like behavioral phenotype in the offspring^12–14^. In addition, these results indicate that factors altering the maternal gut microbiome could also increase the risk for neurodevelopmental abnormalities in the offspring.

Dietary habits are amongst the most important factors shaping the gut microbiome^15,16^. A diet rich in salt (i.e. high salt diet, or HSD) for instance, can result in depletion of the gut bacteria *Lactobacillus* spp.^17^, which results in T helper cell-mediated immune dysregulation^17,18^, and cognitive dysfunction^19–21^. Increased salt intake is a worldwide well-established cause of morbidity and mortality associated with hypertension, cardiovascular diseases as well as kidney failure^22^. However, HSD has not been fully addressed in the context of gut microbiome-mediated prenatal effects on offspring neurodevelopment. Therefore, we report that 8-week chronic consumption of HSD alters the parental gut microbiome composition. Specifically, we found that HSD was associated with depletion of *Lactobacillus* spp. and enrichment of *Akkermansia* in mice fecal samples. We also observed that this altered gut microbiome is transferred to the offspring, which shared the same gut microbial composition with their parents until (at least) their weaning age. Finally, we found that this “inherited” modified gut microbiome is associated with abnormal social behaviors and hyperactivity in adult male mice only, not in females. Together, our results support the notion that ASD-like behaviors can be linked to parental HSD-induced gut dysbiosis.

## Methods

### Animals

Male and female wild-type C57Bl/6 mice (3–4 weeks‐old) were purchased from The Jackson Laboratory (Bar Harbor, ME, US), and housed at Texas Tech University’ animal facility. Mice were group housed (4–5 same-sex mice/cage) in ventilated cages in a temperature‐controlled room (21–23 °C) with 12:12 h light/dark light cycle (lights on 07:00–19:00), humidity of 40–60%, and food and water available ad libitum. All experiments and procedures were performed observing the Animal Research: Reporting of In Vivo Experiments (ARRIVE) guidelines and following relevant guidelines and regulations approved by the Texas Tech University Institutional Animal Care and Use Committee (IACUC).

Mice were randomly assigned to control or HSD groups and were fed chow supplemented with 0.1% NaCl (Control, Teklad Custom Diet TD-94268) or 8% NaCl (HSD, Teklad Custom Diet TD-180241) respectively for 8 weeks. Both diets contained ~ 23% calories from protein, 62% from carbohydrate and ~ 15% from fat. The HSD groups were also given 1% NaCl in their drinking water whereas the control groups drank regular tap water. After 8 weeks male and female mice were used for behavioral assays while others were paired for breeding. All the experiments were performed in a separate room adjacent to the room where mice were housed.

### Behavioral analysis

#### Open field test (OFT)

The open field test was done using a large plastic container measuring 12in width × 18in length × 12in height. Individual mice were placed inside and allowed to freely explore the arena for 5 min. The session was videotaped and then analyzed using Ethovision XT tracking system, which was used to analyze all behavioral tests (Noldus, Leesburg, VA). Several parameters were quantified: Total distance traveled, speed of movement and time spent in the center versus periphery regions. Additionally, the number of rearing events and cumulative grooming time were measured. This test was done on both parents and offspring.

#### Elevated plus maze test (EPM)

The EPM test was performed using a four-arm plus shaped maze elevated 30 inches from the ground, with two arms with walls and two arms open. The arm length was ~ 20 inches with a 4-inch center square between all four arms. Individual mice were placed in the center part and then were allowed to freely explore for 5 min while the session was videotaped. The time the mouse spent in the closed arms versus the open arms was quantified for detecting avoidance behavior^23^.

#### Novel object recognition (NOR) test

The NOR test was performed in the same plastic container used for OFT. The test consisted of two consecutive sessions, a first session used for familiarization and a second session used as a test for memory acquisition. In the familiarization session, two identical objects were placed in the bottom of the container, equidistant from each other and the side walls. Then a single mouse was placed in the arena and allowed to freely explore for 5 min. After this session, the mouse was returned to its home cage. For the test session one of the identical objects was replaced with a novel unfamiliar one. The test session was performed in two phases. For evaluating short term memory, the mouse was placed inside the container 1 h after familiarization. For long term memory, the mouse was placed inside the container 24 h after the familiarization session. In both phases, each mouse was allowed to explore the objects for 5 min while the session was videotaped. The time each mouse spent exploring the familiar and novel objects was quantified and a preference index was calculated using the following equation PI = [time spent exploring novel object/(time exploring familiar object + time exploring novel object)] × 100%^24^.

#### T-maze test

The T-maze test was performed using a T shaped maze, 30 inches elevated from the ground. The total length of the top arm was 44 inches and the bottom arm was 20 inches. All arms had walls. For the test, a single mouse was placed in the outermost corner of the bottom arm and was allowed to explore the whole maze for 5 min while videotaping the session. Working memory was evaluated by quantifying the number of spontaneous alternations between entering the three different arms^25^.

#### 3-chamber sociability test

The 3-chamber sociability test was done in the offspring generation to evaluate possible sociability deficits^26^. The test was done in a plastic container (15in × 19in × 10in) divided in three separate chambers by two inner partitions which had a single small opening to allow mice to move between chambers. The experiment was performed in two consecutive sessions, the first for training and the second for testing. In the training session, a small metal mesh box was placed in both the left and right chambers. An individual subject mouse was placed in the middle chamber and was allowed to explore the entire container for 5 min, then removed and kept in an empty cage while the container was prepared for the following session (test). For the testing session, an unfamiliar mouse (i.e. not a cage mate) of the same sex was placed inside one of the metal mesh boxes and kept there as “social (S) chamber”. The mesh box in the other lateral chamber was kept empty and was referred to as a “non-social (NonS) chamber”. The subject mouse was then placed back in the middle chamber and allowed to explore for 5 min while videotaping. We then quantified the total time that the subject mouse spent interacting with the metal wire container in either the S or NonS chambers. Interaction was defined as sniffing and/or touching the metal wire container while actively exploring it.

#### Marble burying test

The marble burying test was used to evaluate repetitive behaviors in the offspring^27^. For the test we used a clean, empty mouse cage and filled it with to 2 inches of regular bedding material. On top of this layer of bedding, 40 small black marbles were placed in a grid-like organized pattern and a photograph was taken. An individual mouse was placed inside the cage and was allowed to explore for 15 min. After the exploration period, the mouse was returned to its home cage and a second photograph was taken. The number of buried and unburied marbles was quantified by two experimenters blind to the mouse treatment, by comparing the before and after photographs. We considered a single marble as buried if at least 75% of its shape was clearly covered by bedding material^27^.

#### Urinary pheromone test

The urinary pheromone test was performed in the same container used for the OFT. We first collected urine from mice unfamiliar to the experimental groups (i.e. not cage mates) and kept it frozen. Right before the experiment, an aliquot of urine was thawed, a cotton swab was soaked in it and placed at the bottom of the container. Then a single mouse was placed inside and was allowed to explore for 5 min while videotaping the session. We measured the time directly interacting with the swab^28^.

### Fecal sample collection and 16s rRNA Illumina sequencing

Colonic fecal samples were collected from the dams of both diet groups before weaning. The fecal samples from the offspring groups were collected before weaning at 2–3 weeks of age (i.e. while they were still housed with their mothers). To reduce stress, mice were placed inside autoclaved plastic boxes where they freely defecated. Feces were then collected and placed in autoclaved cryotubes, flash frozen in liquid nitrogen and immediately stored at − 80 °C. DNA extraction from the samples, Polymerase Chain Reaction (PCR) amplification of the variable 4 (V4) region of the 16s rRNA gene and sequencing using the Illumina MiSeq platform were performed by the Molecular Research LP laboratories (MR DNA, Shallowater, TX, USA).

### Sequencing data processing and analysis

The data obtained from sequencing was processed using the MR DNA data processing pipeline (MR DNA, Shallowater, TX, USA). Sequences shorter than 150 bp, sequences with ambiguous base call and sequences depleted of barcodes and primers were removed. Operational taxonomic units (OTUs) were defined by clustering at 97% similarity followed by the removal of chimeras. OTUs were taxonomically classified using BLASTn against a curated database derived from RDPII and NCBI. All samples were run concurrently by blinded experimenters. Raw data have been deposited under the umbrella BioProject: PRJNA713927. Data are publicly accessible at https://www.ncbi.nlm.nih.gov/bioproject/PRJNA713927.

### Blood sample preparation

After weaning, female mice from the parental generation were anesthetized with isoflurane and euthanized by decapitation. Trunk blood samples were rapidly collected in autoclaved tubes, and the serum was separated by centrifugation (3000 rpm for 30 min). Proinflammatory cytokines IL-1β, IL-10, IL-17, IL-23, and TNF-α were detected using a LEGENDplex Mouse Inflammation Panel (Biolegend, CA, USA).

### Statistical analysis and data availability

All behavioral analysis was done using Ethovision XT software (Noldus, Leesburg, VA). Data analysis and statistical analysis was done using Origin 2017 software (OriginLab Corporation). For determining statistical differences between two groups, paired and unpaired Student’s t-test was used. Whereas difference between multiple groups was performed by one-way ANOVA followed by Tukey’s post-hoc test. Differences between groups were considered statistically significant at *p* < 0.05. The datasets from behavioral experiments generated during the current study are available in the DRYAD repository https://datadryad.org. The following link can be used to access it: https://datadryad.org/stash/share/PNwbYbu9LheZuwICm11V91LCibtfEqjnR1an1VIRPCE.

## Results

### HSD negatively regulates body weight in male mice and does not impair reproduction

**Figure 1:**
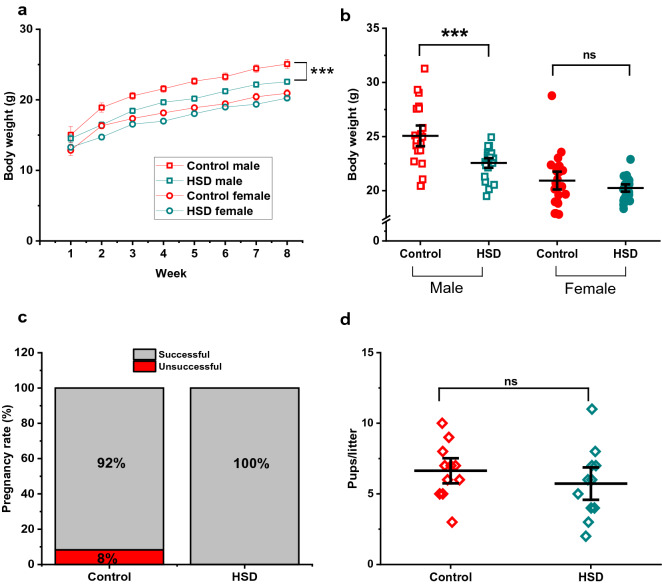
HSD negatively regulates weight in male mice and does not result in reproductive changes in the parental generation. Body weight was measured weekly during the 8-week feeding protocol. (**a**) Each group shows a steady growth although HSD males were significantly smaller (n = 22 control male group, 21 HSD male group, 21 control female group, 22 HSD female group). (**b**) Average body weight at the end of feeding protocol per group. Reproductive health was measured by calculating pregnancy rate (**c**) and quantifying the number of pups per litter (**d**) in each pair (n = 14 control pairs, 15 HSD pairs). Both parameters show that HSD does not alter reproduction ability. Data are described as mean ± SEM. *p < 0.05, **p < 0.01, ***p < 0.001, unpaired t-test (**d**), one-way ANOVA and Tukey’s test (**b**).

To monitor growth during HSD, we weighed mice weekly. Figure 1a shows that over time, HSD-fed male mice gained significantly less weight than controls, while females did not show any significant difference. On average, control male mice weighed 25.07 ± 0.63 g after 8 weeks of HSD, while HSD-fed male mice weighed 22.56 ± 0.29 g (Fig. 1a,b, n = 22/21 control/HSD males; F_(3,82)_ Value = 22.45, Prob > F = 1.05E−10, ***p = 9.68E−4, q-Value = 5.57). Female mice, however, had comparable body weights (female control weighed 20.93 ± 0.54 g, HSD-fed female weighed 20.2 ± 0.22 g; Fig. 1a,b, n = 21/22 control/HSD females; *p* = 0.704, q-Value = 1.52).

After our 8-week feeding protocol a subset of mice was paired to breed, control-fed males and females (n = 14 pairs), or HSD-fed males and females (n = 15 pairs). We kept each breeding pair fed with the same food of their feeding protocol until their offspring reached weaning age. We did not observe any significant difference in fertility (Fig. 1c, 92% pregnancy rate in control-fed vs. 100% in HSD-fed pairs, *p* = 0.37, unpaired T-test, t-score = 1.414), as well as number of pups born per litter (Fig. 1d, 6.64 ± 0.59 pups/litter in control vs. 5.73 ± 0.76 pups/litter in HSD pairs, *p* = 0.358, unpaired T-test, t-score = 0.941). Overall, these results indicate that 8 weeks of HSD do not significantly impair reproduction in mice.

### HSD protocol does not alter exploratory behavior or memory tasks performance

**Figure 2:**
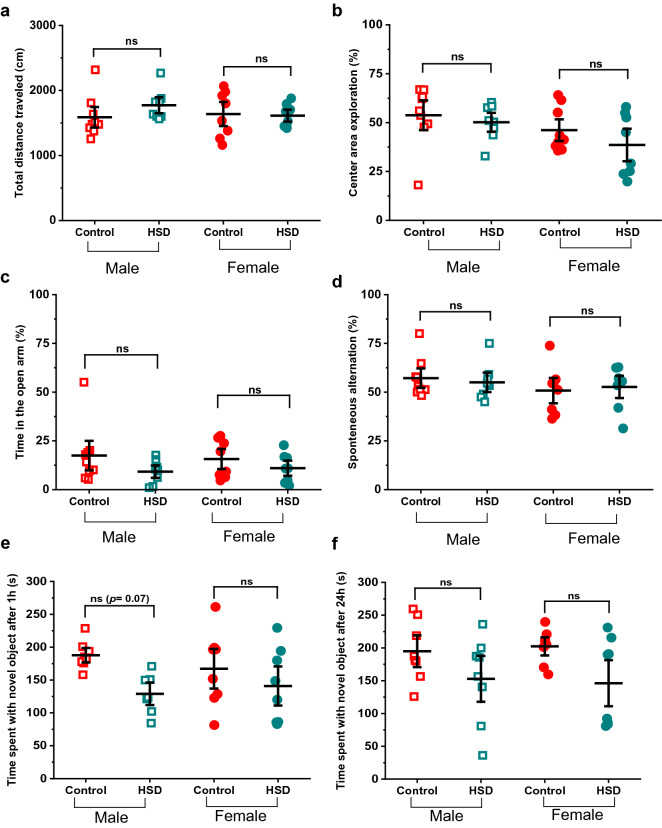
HSD does not alter exploratory behavior or memory tasks performance in the parental generation. The behavioral tests used were open field test (OFT), elevated plus maze (EPM), T-maze spontaneous alternation test and novel object recognition (NOR) test (n = 9 control males, 8 HSD males, 8 control females, 8 HSD females). Overall, no difference was observed in exploratory behavior in HSD-fed groups: (**a**) total distance traveled in the OFT, (**b**) time spent in the center area of the OFT, or open arm exploration in EPM (**c**). In addition, HSD-fed mice do not show memory impairment in the T-maze spontaneous alternation test (**d**), or in the NOR test after 1 h (**e**, although there is a clear trend toward reduced memory in HSD-fed male mice but it did not reach statistical significance). NOR test after 24 h shows similar results, no difference in memory performance (**f**). Data are described as mean ± SEM. *p < 0.05, **p < 0.01, ***p < 0.001, one-way ANOVA and Tukey’s test.

To investigate the direct effects of HSD on exploratory behaviors and memory function, we performed a series of well-established behavioral assays: open field test (OFT), elevated plus maze (EPM), novel object recognition (NOR) test and T-maze alternation test. Figure 2a shows the average total distance traveled in the OFT for all groups, where no significant differences were found (control-fed males n = 9, total distance = 1587.39 ± 104.99 cm; HSD-fed males n = 8, total distance = 1773.04 ± 82.74 cm, *p* = 0.53, q-Value = 1.94; control-fed females n = 8, total distance = 1637.82 ± 123.31 cm; HSD-fed females n = 8, total distance = 1612.77 ± 61.35 cm, *p* = 0.99, q-Value = 0.25; F_(3,29)_ Value = 0.73, Prob > F = 0.54). Furthermore, we calculated the percentage of time spent in the center area (%center) of the OFT arena (Fig. 2b) and found no difference between HSD and control-fed mice as well (%center in control males = 53.79 ± 5.05% vs. HSD-fed males = 50.18 ± 3.22%; F_(3,29)_ Value = 2.03, Prob > F = 0.13, *p* = 0.94, q-Value = 0.79; control-fed females = 47.43 ± 3.92%; HSD-fed females = 38.56 ± 5.55%; *p* = 0.53, q-Value = 1.90). Similarly, while there was a slight tendency towards HSD-fed groups spending less time in the EPM open arms, the statistical analysis showed no statistically significant difference compared to the control-fed groups (Fig. 2c, time spent in the open arms in control male = 17.5 ± 5.05%; HSD-fed male = 9.2 ± 2.11%, *p* = 0.38, q-Value = 2.31; control-fed females = 15.7 ± 3.42; HSD-fed females = 11.0 ± 2.58, *p* = 0.804, q-Value = 1.275; F_(3,29)_ Value = 1.16, Prob > F = 0.34).

In addition, we evaluated working memory using the T-maze alternation test, and spatial memory using the NOR test (both short- and long-term). Figure 2d shows that control and HSD-fed mice displayed a similar performance in the T-maze. On average, the spontaneous alternation in the control-fed male group was 57.15 ± 3.32% versus 55.03 ± 3.31% in the HSD-fed male group (*p* = 0.98, q-Value = 0.58; F_(3,29)_ Value = 0.58, Prob > F = 0.63). Comparably, the control-fed females showed 50.78 ± 4.33% alternation versus 52.65 ± 3.81% in the HSD-fed group (*p* = 0.98, q-Value = 0.49). In the NOR test we quantified the time that mice spent exploring the novel object after either 1 or 24 h post-training period (Fig. 2e,f, see “Methods” section for specific details). We first compared this value to the 50% chance between both objects (i.e. 150 out of 300 s total time). Male mice fed with control diet spent on average 187.8 ± 7.35 s interacting with the novel object during the NOR test after 1 h of training (*p* = 0.00134, One sample t-test), while females spent 167.3 ± 20.1 s which was not statistically different from 50% chance (*p* = 0.418, One sample t-test). The HSD-fed group did not show significant impairment in either short- or long-term term memory formation, although there was a clear trend to reduced short-term performance in males (Fig. 2e). On average control-fed male mice spent 187.8 ± 7.35 s exploring the novel object 1 h after the training session, compared to 129.01 ± 11.43 s in the HSD-fed group (*p* = 0.07, q-Value = 3.62; F_(3,27)_ Value = 2.2.71, Prob > F = 0.06). Similarly, female mice fed with HSD spent 140.88 ± 19.86 s exploring the novel object compared to 167.3 ± 20.1 s in the control fed group (*p* = 0.64 vs. control-fed, q-Value = 1.68). Comparable results were observed 24 h after the training session (Fig. 2f). As done with the 1 h NOR test, we first compared the control value with the 50% chance between both objects. Male mice fed with control food spent on average 194.95 ± 16.13 s interacting with the novel object after 24 h (*p* = 0.027, One sample t-test), while females spent 202.43 ± 9.21 s interacting with the novel object (*p* = 0.0074 vs. 50% chance, One sample t-test). When comparing with HSD-fed mice, we found that control-fed male mice spent 194.95 ± 16.13 s exploring the novel object versus 152.88 ± 23.31 s in the HSD-fed male group (*p* = 0.41, q-Value = 2.22; F_(3,29)_ Value = 2.29, Prob > F = 0.10). Female mice showed comparable results. Control-fed female mice spent 202.43 ± 9.21 s versus 146.20 ± 23.41 s observed in the HSD-fed group (*p* = 0.18, q-Value = 2.97). Taken together, and consistent with previous reports^19^, our results show that 8-week of HSD did not significantly alter the measured behaviors in our mice.

### Altered gut microbiome found in HSD-fed parental female mice is shared with their offspring

Maternal microbiome can transfer to the offspring during birth^6^. It has also been shown that elevated salt consumption can lead to changes in the maternal gut microbiome composition^17,18^. Therefore, we reasoned that it is possible that our feeding protocol could be altering the maternal gut microbiome and that this microbiome could be then transferred to the offspring. To test this hypothesis, we collected fecal samples from females in the parental group (i.e. from both control and HSD-fed mothers or dams) and their offspring mice (i.e. at 2–3 weeks old, before weaning). We then performed 16 s rRNA amplicon sequencing (see “Methods” section for details) to obtain an accurate description of their gut microbiome composition.

**Figure 3:**
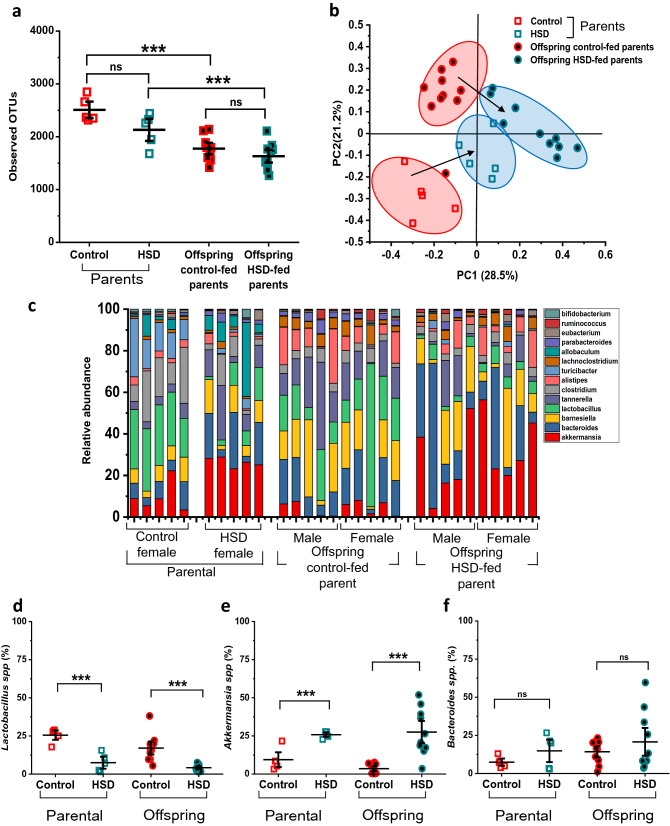
Altered gut microbiome found in HSD-fed parental female mice is shared with their offspring. Gut microbiome data was obtained from fecal samples and analyzed from 16 s rRNA sequencing. (**a**) No difference was found in observed OTUs between control and HSD-fed dams as well as between their respective offspring (n = 5 control-fed dam, 5 HSD-fed dam, 10 mice from control-fed parent, 10 from HSD-fed parent). (**b**) Principal coordinate analysis (PCoA) plots of Bray–Curtis distance metrics show separate clustering of microbiome from parental and offspring generation. Black arrows indicate the direction of significant shift in microbiota composition between groups. (**c**) Summary of relative abundance of the top 14 most abundant microbial genera in the community sampled. Topmost abundant genera, *Lactobacillus spp* was significantly reduced, *Akkermansia spp* was increased and *Bacteroides spp* was unchanged in HSD-fed dams as well as in their offspring (**d**, **e**, **f**). Data are described as the mean ± SEM. *p < 0.05, **p < 0.01, ***p < 0.001, one-way ANOVA and Tukey’s test.

Results in Fig. 3a show that alpha-diversity (calculated based on observed operation taxa units or OTUs) was not significantly different, although there was a trend towards reduction in HSD-fed dams. The average OTUs observed in the control-fed group of dams was 2508.2 ± 104.74 versus 2129.6 ± 139.41 in HSD-fed counterparts (n = 5 in both control/HSD-fed, *p* = 0.11, q-Value = 3.34, F_(3,26)_ Value = 15.55, Prob > F = 5.41E−6). Similarly, we found no differences between offspring mice. The average OTUs in the offspring from control-fed parents was 1775.2 ± 72.72 versus 1630.9 ± 80.50 in offspring from HSD-fed groups (total n = 10 for both groups, 5 females and 5 males in each; *p* = 0.59, q-Value = 1.801). However, we found a significant reduction in observed OTUs when comparing dams to their respective offspring (Fig. 3a, Control-fed dam vs. offspring from control-fed dam, ***p = 8.80E−5, q-Value = 7.47; HSD-fed dams vs. offspring from HSD-fed parents **p = 0.006, q-Value = 5.08). Further, our microbiome data analysis showed a significant difference in beta diversity between parental HSD and control-fed samples as well as their offspring (Fig. 3b), based on the non-phylogenetic Bray Curtis distance matrix (ADONIS of Bray Curtis: R^2^ = 0.48, F_(3,26)_ Value = 7.86, **Prob > F = 0.001)^29^. The PCoA graph shows that data points from HSD-fed dams were closely clustered, indicating comparable microbial diversity. Moreover, when comparing this group with control-fed dams, we observed that while control-fed mice also clustered together, they did so significantly far from the HSD-fed parental group. Lastly, the offspring from HSD and control-fed dams were also clustered in distance from each other whereas HSD-fed parents and their offspring are clustered in closer proximity (Fig. 3b).

Based on relative abundance, we listed the 14 most represented bacterial genera in the gut microbiome of both parental and offspring generations (Fig. 3c) and observed that the abundance of specific bacterial genera was significantly altered. Further, the genus *Lactobacillus* showed the greatest decrease in dams after 8 weeks on HSD compared to control-fed females, thus confirming previous findings^17,18,30^. On the contrary, the genus *Akkermansia* showed a robust increase in HSD-fed groups. Our data shows two important results. First, there were clear differences in the abundance of specific bacterial genera in dams fed with either control or HSD. Second, those differences were clearly maintained between parents and offspring mice fed with the same diet, which supports the notion of mother-to-infant microbial transmission^31,32^. Furthermore, we specifically analyzed the presence of *Lactobacillus* spp. and found that these bacteria were significantly reduced in both HSD-fed dams and their offspring (Fig. 3d). Our analysis showed that 25.54 ± 2.04% of the total bacterial abundance in the control-fed dams was *Lactobacillus* spp whereas in the HSD-fed parental groups this was reduced to 7.47 ± 2.62% (F_(3,26)_ Value = 16.41, Prob > F = 3.47E−6, ***p = 4.96E−4, q-Value = 6.54). The offspring groups showed similar differences in bacterial abundance: % of *Lactobacillus* spp in offspring from control-fed dams = 17.08 ± 2.8% whereas in the offspring from HSD-fed dams this % was 4.18 ± 0.61% (***p = 4.41E−4, q-Value = 6.60). On the contrary, *Akkermansia* spp. was found significantly increased in the HSD-fed dams and their offspring. As shown in Fig. 3e, *Akkermansia* spp % in control-fed dams was 9.19 ± 3.21% compared to 25.79 ± 0.95% in the HSD-fed parental group (F_(3,26)_ Value = 12.76, Prob > F = 2.55E−5, ***p = 0.001, q-Value = 6.90). The offspring groups showed a similar increase in *Akkermansia* spp: % in offspring from control-fed dams = 3.52 ± 1.02% versus 27.52 ± 4.84% found in offspring mice from HSD-fed dams (***p = 4.44E−5, q-Value = 7.85). Our microbiome analysis also showed no difference in the bacterial abundance content of *Bacteroides* spp (Fig. 3f). The % of *Bacteroides* spp in control-fed dams was 7.42 ± 1.59% whereas in the HSD-fed females this % = 14.84 ± 4.90% (F_(3,26)_ Value = 1.22, Prob > F = 0.32, *p* = 0.80, q-Value = 1.28). Consequently, the offspring groups showed similar results. The % of *Bacteroides* spp in the offspring of control-fed dams = 14.73 ± 2.38% vs. 20.73 ± 6.08% observed in the offspring from HSD-fed dams (*p* = 0.68, q-Value = 1.57).

Overall, our data also suggests that as the maternal gut microbiome is likely transferred to the offspring during the first postnatal weeks, it provides a route for shaping the initial microbial population in the offspring that could be altered according to the maternal diet.

### HSD does not change serum concentration of proinflammatory cytokines in female parental mice

**Figure 4:**
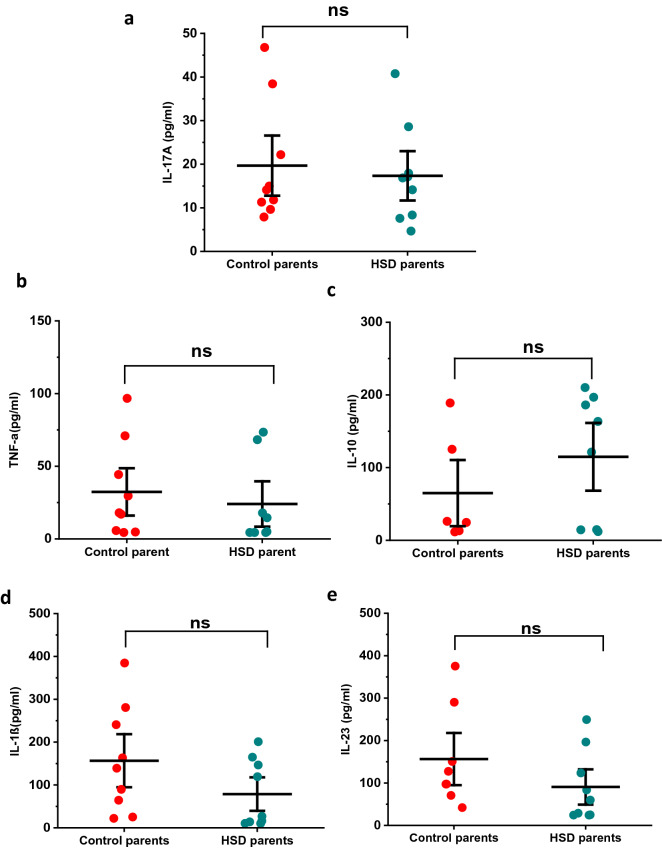
HSD does not change serum concentration of proinflammatory cytokines in female parental mice. Serum concentration on proinflammatory cytokines were measured using flow cytometry-based assay after offspring weaning. (**a**, **b**) Samples from HSD-fed female mice showed no change in the concentration of proinflammatory cytokines IL-17 (n = 9 in control and HSD-fed group) and TNF-α (n = 9 in control and n = 8 in HSD-fed group), or the (**c**) anti-inflammatory cytokine IL-10 (n = 6 control, n = 8 HSD-fed). (**d**, **e**) Proinflammatory cytokines IL-1β (d, n = 9 in control and HSD-fed group) and IL-23 (**e**, n = 8 in control and n = 9 in HSD-fed group) were not significantly reduced in HSD-fed females (n = 5 and n = 4 control, 5 HSD). Data are described as mean ± SEM.

Previous studies have shown that HSD can induce immune system dysregulation in mice^19–21^. Therefore, we collected trunk blood samples from a group of dams after several weeks in HSD or control diet and measured specific cytokine levels. Blood collection was carried out at the time litters were weaned at postnatal day 28. Figure 4a shows that in HSD-fed mice the IL-17 concentration was not different from control-fed mice. On average we measured 19.68 ± 4.58 pg/ml in control versus 17.33 ± 3.77 pg/ml in HSD-fed mice (control, n = 9, HSD, n = 9, *p* = 0.60, t-score = 0.39, unpaired t-test). In addition, we measured the concentration of IL-1β, IL-23, TNF-α, and IL-10. Similar to IL-17, neither TNF-α nor IL-10 were different between diet treatments (Fig. 4b–c). We measured 32.37 ± 10.84 pg/ml of TNF-α in control versus 24.04 ± 10.39 pg/ml in HSD-fed mice (control n = 9, HSD n = 8, p = 0.50, t-score = 0.55). IL-10 was 64.91 ± 30.38 pg/ml in control versus 114.83 ± 31.06 pg/ml in HSD-fed female mice (control, n = 6, HSD, n = 8, *p* = 0.28, t-score = − 1.12). Furthermore, we found that while there was trend towards reduced values, IL-1β and IL-23 were not statistically reduced in HSD-fed dams (Fig. d–e). In fact, the concentration of IL-1β was 156.62 ± 41.35 pg/ml in control versus 78.73 ± 26.04 pg/ml in HSD-fed female mice (control, n = 9, HSD, n = 9, *p* = 0.13, t-score = 1.60). IL-23 concentration was 156.43 ± 40.00 pg/ml in control and 90.65 ± 27.73 pg/ml in HSD-fed female mice (control, n = 8, HSD, n = 9; *p* = 0.19, t-score = 1.36). Taken together, these results indicate that while our HSD protocol resulted in altered gut microbiome composition, it did not increase the serum concentration of proinflammatory molecules.

### Adult male offspring from HSD-fed mice show hyperactivity, reduced sociability and increased repetitive behaviors

We investigated potential long-term effects of parental HSD on their offspring behavior, focusing on exploration, repetitive and social behaviors. At the time of weaning, we transitioned all offspring mice to control diet and regular water (i.e. no added NaCl) to avoid direct effects of diet on their behavior.

**Figure 5:**
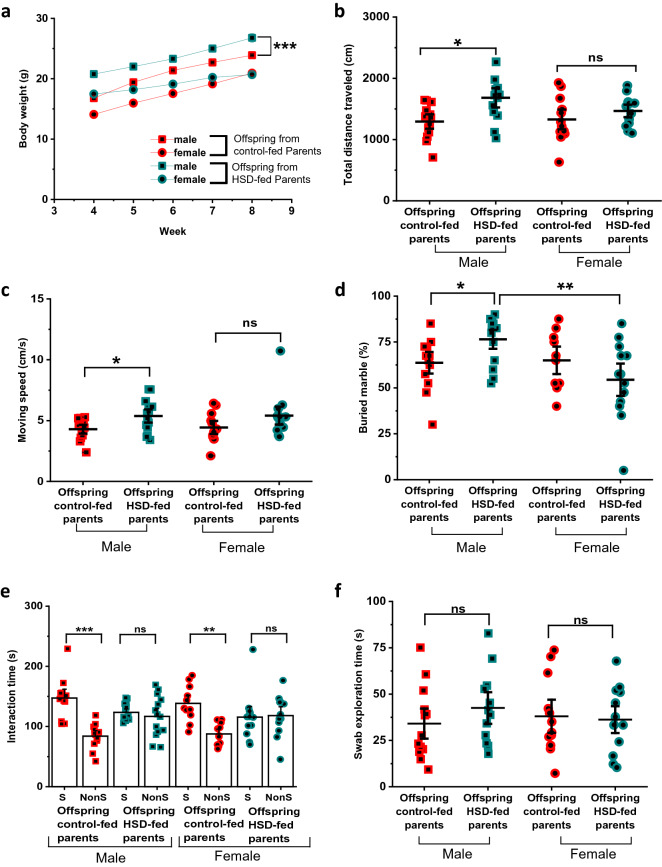
Male offspring from HSD-fed mice are heavier and show ASD-like behavioral phenotype. Body weight of the offspring generation was measured from weaning to adulthood. (**a**) Male and female offspring from HSD-fed parents weigh significantly higher at the age of weaning and only male mice continued to weigh more until their 8th week (n = 30 in all groups). (**b**, **c**) Male offspring from HSD-fed parents show higher distance traveled and moving speed in the OFT (n = 13 male offspring from control-fed parents, 14 male offspring from HSD-fed parents, 12 female offspring from control-fed parents, 13 female offspring from HSD-fed parents). In the marble burying test, male offspring from HSD-fed parents show higher repetitive behavior (**d**). Offspring from HSD-fed parents show lower preference for the Social (S) chamber in the 3-chamber test compared to offspring from control-fed parents, (**e**) but no difference was observed in the urinary pheromone test (**f**). Data are described as mean ± SEM. *p < 0.05, **p < 0.01, ***p < 0.001, one-way ANOVA and Tukey’s test.

We first monitored body weight weekly from their weaning age until 8–10 weeks old when behavioral experiments were conducted. At 4-weeks old, both male and female offspring from HSD-fed parents were significantly heavier than offspring from control-fed pairs. However, the difference in body weight persisted only in males (Fig. 5a). At 4 weeks old the average weight in males from control-fed parents was 18.39 ± 2.02 g versus 20.44 ± 1.79 g in male offspring from HSD-fed mice (Fig. 5a and Supplementary Figure 1a, n = 30 in all groups; F_(3,115)_ = 53.13, Prob > F = 1.27E−21, ***p = 1.17E−4, q-Value = 6.29). Similarly, female mice from control-fed parents weighed on average 14.69 ± 1.44 g versus 17.34 ± 1.81 g from females from HSD-fed parents (n = 29/30 for offspring from control/HSD-fed parents, ***p = 5.19E−7, q-Value = 8.08; male vs. female offspring from Control-fed parents ***p < 1E−12, q Value = 11.28; male vs. female offspring from HSD-fed parents ***p < 1E−12, q-Value = 9.52). Moreover, at 8 weeks old the average weight in males from control-fed dams was 23.89 ± 0.56 g versus 26.66 ± 2.10 g in male offspring from HSD-fed mice (Fig. 5a and Supplementary Figure 1b, n = 16 in all groups; F_(3,60)_ = 90.75, Prob > F = 2.86E−22, ***p = 5.38E−8, q-Value = 9.36). Similarly, female mice from control-fed parents weighed on average 20.92 ± 0.60 g versus 20.72 ± 0.68 g from females from HSD-fed parents (*p* = 0.97, q-Value = 0.65; male vs. female offspring from Control-fed dams ***p < 1E−12, q-Value = 2.13; male vs. female offspring from HSD-fed dams ***p < 1E−12, q-Value = 2.13).

Previous research has shown that altered gut microbiome is associated with behavioral signs of ASD^33,34^. The offspring from our HSD-fed parents showed altered gut microbiome during juvenile stages, thus we hypothesized that these changes might lead to abnormal behavior. We therefore characterized social and exploratory behaviors in offspring from control and HSD-fed mice using the OFT. Figure 5b shows that male offspring from HSD-fed parents explored significantly larger area. On average, male mice from control-fed parents moved 1293.94 ± 77.99 cm versus 1640.09 ± 105.85 cm covered by male offspring from HSD-fed parents (n = 13/14 for male offspring from control/HSD-fed parents; F_(3,48)_ = 3.06, Prob > F 0.03, *p = 0.04, q-Value = 3.85). These differences, however, were not observed in females. The area covered by females from control-fed parents was 1328.07 ± 107.35 cm versus 1468.10 ± 67.55 cm in females from HSD-fed parents (n = 12/13 for female offspring from control/HSD-fed parents; *p* = 0.71, q-Value = 1.49). Furthermore, male offspring from HSD-fed parents moved faster (Fig. 5c). The average speed in males from control-fed parents was 4.14 ± 0.90 cm/s versus 5.52 ± 1.22 cm/s in male from HSD-fed parents (F_(3,48)_ = 3.58, Prob > F = 0.02, *p = 0.04, q-Value = 3.83). These differences were again not statistically different between female offspring (speed in females from control-fed parents = 4.43 ± 1.24 cm/s; speed in females from HSD-fed parents = 5.41 ± 1.78 cm/s; *p* = 0.27, q-Value = 2.58). We also quantified other behaviors such as avoidance (i.e. time in the center of the OFT arena), rearing and grooming (Supplementary Figure 2) and found no significant differences between offspring from control and HSD-fed parents. These results indicate that male offspring from HSD-fed mice show hyperactivity, one of the prominent signs of ASD-like behavior in mouse models^35^.

To characterize repetitive behavior we used the marble burying test^27^. We found that only male offspring from the HSD-fed parents buried a significantly higher % of marbles compared to male mice from control-fed parents. Figure 5d shows that, on average, male mice from control-fed parents buried 59.81 ± 4.10% of marbles versus 77.14 ± 3.14% in male mice from HSD-fed parents (F_(3,46)_ = 5.03, Prob > F = 0.004; *p = 0.04, q-Value = 3.93). Female mice from the control-fed group buried 65 ± 4.99% of marbles compared to 54.42 ± 5.87% in female mice from HSD-fed parents (*p* = 0.41, q-Value = 2.19).

We also evaluated social behavior in the offspring generation because it has been shown to be modulated by the commensal microbiome, and deficits in social behavior are key signs of ASD^36,37^. Therefore, we subjected our mice to a 3-chamber sociability test (Fig. 5e) and to the urinary pheromone social communication test (Fig. 5f). In the 3-chamber test we quantified the time mice spent exploring the social (S) versus the non-social (Non-S) chamber. Figure 5e shows that the male offspring from HSD-fed parents showed no preference for the social chamber compared to the male offspring from the control-fed group that spent more time in the S chamber versus NonS. On average, the male offspring from control-fed parents spent 147.31 ± 9.85 s in the S chamber compared to 84.06 ± 6.06 s in the NonS chamber (F_(3,48)_ = 11.09, Prob > F = 0.000012; ***p = 0.0000042, q-Value = 8.06). Conversely, male offspring from HSD-fed parents spent 123.44 ± 3.94 in the S chamber compared to 116.69 ± 9.07 s spent in the NonS chamber (*p* = 0.91, q-Value = 0.93). Female offspring from HSD-fed parents showed similar social behavior. Female offspring from control-fed parents spent 138.45 ± 9.02 s in the S chamber versus 87.59 ± 5.47 s in the NonS chamber (F_(3,42)_ = 4.67, Prob > F = 0.0066; **p = 0.0032, q-Value = 5.26). Female offspring from HSD-fed parents on the other hand spent 115.79 ± 11.68 s in the S chamber versus 117.97 ± 9.92 s in the NonS chamber (*p* = 0.99, q-Value = 0.24).

In the urinary pheromone test (Fig. 5f), all groups behaved comparably, and no statistically significant difference was observed. On average, the % of time interacting with the urine-soaked swab in male mice from control-fed parents was 34.08 ± 5.38% versus 42.51 ± 5.69% in male mice from HSD-fed parents (*p* = 0.69, F_(3,48)_ = 0.43, Prob > F = 0.73; q-Value = 1.54). Likewise, the time interacting with the urine-soaked swab in female offspring from control-fed parents was 38.04 ± 5.96% versus 36.22 ± 4.83% in female offspring from HSD-fed parents (*p* = 0.99, q-Value = 0.29).

Altogether our results show important sex-dependent long-term effects associated with parental HSD. Specifically, male offspring from HSD-fed parents showed hyperactivity, social preference deficits and increased repetitive behavior. Interestingly, these alterations closely resemble other animal models of ASD^36^ suggesting the possibility that HSD could be an environmental risk factor for neurodevelopmental disorders.

## Discussion

In this study we showed that: (1) the altered parental microbiome is shared with their offspring (likely transmitted during birth and first postnatal weeks), (2) these changes can be detrimental for neurodevelopment, and (3) altered gut microbiome is associated with ASD-like behavioral abnormalities in the offspring adulthood, changes that are only visible in male mice.

Previous studies have shown that HSD can modulate body weight, however, the effects have varied from no change, to either increase or decrease^38–40^. These discrepancies have been mostly attributed to differences in experimental design, i.e. salt content (4%, 7% or 8% in different studies), feeding period length (2, 4, 7 or 8 weeks) or nutritional differences between diets, most notably different fat content (10, 14 or 60%)^40–43^. Our results showing that HSD-fed male mice had a lower body weight replicate previous findings and agree with other studies using similar fat content. Our experiments also expanded these results to females, which were found to not be affected, in another example of sex-dependent differences. Unfortunately, there is limited literature addressing response to HSD in female mice. A recent study in female rats showed that HSD prevented weight gain caused by a high-fat diet^40^, while causing no effect in low-fat conditions. This reduction in weight gain was associated with adipocyte size and reduction in leptin levels^40^. Our results further emphasize the need for including sex as a biological variable in future studies examining how environmental factors (such as food) influence metabolism and neurodevelopment.

HSD has been associated with cognitive impairment due to several mechanisms including increasing oxidative stress, reducing the expression of synaptic proteins such as synapsin and CamK (Calcium‐calmodulin dependent protein kinase), as well as by reducing the resting cerebral blood flow and nitric oxide production in cerebral endothelial cells^19,44^. While these studies have implemented HSD of varied duration (i.e. 4 to 24 weeks long), they have reported short-term memory impairment after 7 weeks of HSD^19,44^, and long-term memory impairment even after 4 weeks on HSD^44^. Moreover, another study showed that HSD can impair cognitive function in an age dependent manner, namely HSD was associated with impaired cognitive function only in 20-months old rats but not in young adults (2-months old)^45^. In our study, we started HSD feeding at 4-weeks old and after 8 weeks (i.e. at 12 weeks of age), we only observed a trend towards reduced short-term memory function in the NOR test in male mice, but the difference was not statistically significant from the control-fed group. The discrepancy with previous findings might be related to the much younger starting age in our experiments. We also investigated stress and anxiety-related behaviors in HSD-fed mice and found no significant differences, which is in line with previous findings^45^. Taken together, our data indicates that HSD-fed mice were not significantly different from control-fed after 8 weeks of HSD. Importantly, our study also included female mice and showed that females were not affected by HSD (at least under our experimental conditions). Nevertheless, our findings warrant more experiments using female mice.

Dietary habits have been shown to change the gut microbiome composition^16,46,47^. For instance, short-term consumption of entirely animal or plant-based food resulted in completely different gut microbiome community structure in humans^48^. In this context, several recent studies showed that feeding rodents with HSD for 4–8 weeks changed the abundance levels of specific microbial genera^17,18,30^. It has also been found that while beta diversity was significantly different in mice fed with HSD^17^, alpha diversity was not different between HSD and control-fed groups^17,18,30^. Our data were consistent with these results, namely our HSD-fed dams had reduced abundance of *Lactobacillus* and a significant increase in the abundance of genus *Akkermansia* compared to control-fed mice. However, we also found that our HSD-fed mice did not show a comparable increase in proinflammatory cytokines. It has been shown that HSD can alter the immune homeostasis by increasing the production of proinflammatory cytokines such as IL-17, IL-23, IL-1β, TNF-α^19–21^. This difference could be attributed to methodology employed since we performed flow cytometry-based multiplex assay for serum cytokines detection, whereas other studies used flow cytometry for measuring the differentiation of CD4 + T cells into pro-inflammatory T-cells^18,20,21^. However, another possible explanation for the observed lack of increased cytokines could be the abundance of *Akkermansia* spp in the gut of our HSD-fed mice. *Akkermansia *spp is considered as the next-generation probiotic due to its effects on glucose and lipid metabolism, and specially as potent anti-inflammation agent^49,50^. Several studies have shown that *Akkermansia* spp can reduce inflammation by a number of different mechanisms such as reducing plasma level of lipopolysaccharide (LPS)-binding protein (LBP), reducing the expression of inflammatory genes, and reducing the production of pro-inflammatory cytokines such as IL-17, IL-23, IL-8, TNF-α as well as increasing the production of anti-inflammatory cytokines such as IL-10 and IL-12^51–56^. Furthermore, while low levels of *Akkermansia* were found in obesity and type-2 diabetes mouse models, exogenous administration of *Akkermansia* increased the control of gut inflammation and permeability by regulating tight-junction-related proteins, and thickening the intestinal mucous layer^57^. Accordingly, the commonly used diabetes treatment Metformin has been reported to increase *Akkermansia *spp abundance^58^, and to significantly improve glucose metabolism in high-fat diet fed mice while also increasing the number of mucin-producing goblet cells^59^. Moreover, the population of *Akkermansia* in the gut is negatively modulated by the fat content of the consumed diet^54,60,61^. Our HSD has a fat content of approximately 15% which is considered low-fat^62^. Therefore, the apparent lack of inflammation in our HSD-fed mice could originate from the increased presence of *Akkermansia* spp in their gut.

According to several studies, the maternal gut microbiome during pregnancy is vertically transmitted to the offspring during the childbirth and thus it is uniquely poised to shape the early-life microbiome in the newborn^1,6,63^. Initially after birth, the infant microbiome consists mostly of microbes from maternal skin and/or birth canal (according to birth conditions), but the microbes from maternal gut remain more constant in the infant gut^31,32^. In our study, we observed similar changes in microbial composition in the offspring of either control or HSD-fed mice, supporting the idea that the original microbiome is first dictated by the mother’s microbiome, and that it stays relatively unchanged during the first 2–3 postnatal weeks.

Research using germ-free mice has revealed that the gut microbiome present during early developmental stages is crucially important for the nervous and immune system development, brain function and ultimately behavior of the offspring^64^. Further, alterations in the early microbiome linked to maternal diet have been associated with neurodevelopmental disorders such as ASD^65^. For example, a recent study showed that female mice fed with a high fat diet for 8-weeks produce offspring that display several behaviors indicative of ASD^66^. Further, sequencing of the gut microbiome of both dams and their offspring showed significant alterations due to high fat diet, and the behavioral abnormalities were reversed by co-housing the offspring with offspring from control-fed dams^66^. Our data also showed that HSD-fed dams had a different gut microbiome compared to control-fed females. Therefore, we hypothesized that the offspring of HSD-fed mice could indeed display behavioral abnormalities resembling ASD. Our results supported this idea and showed a significant sex-dependent effect: only males consistently performed differently from offspring from control-fed parents. Specifically, the male offspring from HSD-fed parents showed increased locomotion, excessive marble burying as a compulsive behavior and reduced social interaction, all behavioral abnormalities resembling ASD-like phenotype in mice^36,67^.

Though the initial gut microbiome is essential for normal brain development, how the HSD-associated decrease in *Lactobacillus* leads to ASD-like behavioral abnormalities, is still unclear. Gut *Lactobacillus* was found to regulate emotional behavior and GABA expression in mice^68^. Further, the Shank3 KO mice (established ASD model) has reduced gut *Lactobacillus* abundance as well as reduced expression GABA receptors in hippocampus and prefrontal cortex, changes that were reversed by the probiotic administration of a strain of *Lactobacillus* spp^69^. In addition, low levels of *Lactobacillus* were also associated with reduced levels of hypothalamic oxytocin in mice^66^. Our results show that the offspring from HSD-fed parents has reduced level of *Lactobacillus,* most likely passed from their mother. Therefore, we can speculate that low *Lactobacillus* abundance might hamper the expression of GABA receptors and secretion of oxytocin hormone in the male offspring from HSD-fed parents, which ultimately results in ASD-like behavior. More extensive studies are needed to dissect the mechanism underlying the association between low gut *Lactobacillus* abundance and ASD-like behaviors.

## Supplementary information

Supplementary Information 1.

Supplementary Information 2.

Supplementary Information 3.
