# Author Correction: Altered gut microbiome and autism like behavior are associated with parental high salt diet in male mice

**DOI:** 10.1038/s41598-022-10040-2

**Published:** 2022-04-05

**Authors:** Kazi Farhana Afroz, Noah Reyes, Kobe Young, Kajal Parikh, Varsha Misra, Karina Alviña

**Affiliations:** 1 Department of Biological Sciences, Texas Tech University, Lubbock, TX 79409 USA; 2 Honors College, Texas Tech University, Lubbock, TX 79409 USA; 3 Department of Neuroscience, University of Florida, 1395 Center Drive, Room D5-33D, Gainesville, FL 32610-0244 USA

Correction to: *Scientific Reports* 10.1038/s41598-021-87678-x, published online 16 April 2021

The original version of this Article contained errors.

In the Results,

“Figure 5d shows that, on average, male mice from control-fed parents buried 59.81 ± 4.10% of marbles versus 77.14 ± 3.14% in male mice from HSD-fed parents (F_(3,46)_ = 1.68, Prob > F = 0.004; *p = 0.04, q-Value = 3.93).”

now reads:

“Figure 5d shows that, on average, male mice from control-fed parents buried 59.81 ± 4.10% of marbles versus 77.14 ± 3.14% in male mice from HSD-fed parents (F_(3,46)_ = 5.03, Prob > F = 0.004; *p = 0.04, q-Value = 3.93).”

And the text,

“In the urinary pheromone test (Fig. 5f), all groups behaved comparably, and no statistically significant difference was observed. On average, the % of time interacting with the urine-soaked swab in male mice from control-fed parents was 34.08 ± 5.38% versus 51.32 ± 7.71% in male mice from HSD-fed parents (p = 0.19, F_(3,48)_ = 1.68, Prob > F = 0.18; q-Value = 2.85).”

now reads:

“In the urinary pheromone test (Fig. 5f), all groups behaved comparably, and no statistically significant difference was observed. On average, the % of time interacting with the urine-soaked swab in male mice from control-fed parents was 34.08 ± 5.38% versus 42.51 ± 5.69% in male mice from HSD-fed parents (p = 0.69, F_(3,48)_ = 0.43, Prob > F = 0.73; q-Value = 1.54).”

In the Discussion,

“Though the initial gut microbiome is essential for normal brain development, how the HSD-associated increase in Lactobacillus leads to ASD-like behavioral abnormalities, is still unclear.”

now reads:

“Though the initial gut microbiome is essential for normal brain development, how the HSD-associated decrease in Lactobacillus leads to ASD-like behavioral abnormalities, is still unclear.”

In addition, the Statistical analysis and data availability statement,

“All behavioral analysis was done using Ethovision XT software (Noldus, Leesburg, VA). Data analysis and statistical analysis was done using Origin 2017 software (OriginLab Corporation). For determining statistical differences between two groups, paired and unpaired Student’s t-test was used. Whereas difference between multiple groups was performed by one-way ANOVA followed by Tukey’s post-hoc test. Differences between groups were considered statistically significant at p < 0.05. The raw and processed data of all the experiments is available upon request.”

now reads:

“All behavioral analysis was done using Ethovision XT software (Noldus, Leesburg, VA). Data analysis and statistical analysis was done using Origin 2017 software (OriginLab Corporation). For determining statistical differences between two groups, paired and unpaired Student’s t-test was used. Whereas difference between multiple groups was performed by one-way ANOVA followed by Tukey’s post-hoc test. Differences between groups were considered statistically significant at p < 0.05. The datasets from behavioral experiments generated during the current study are available in the DRYAD repository https://datadryad.org. The following link can be used to access it: https://datadryad.org/stash/share/PNwbYbu9LheZuwICm11V91LCibtfEqjnR1an1VIRPCE.”

