# Supplementary material for: Altered gut microbiome and autism like behavior are associated with parental high salt diet in male mice
Source: Sci Rep. 2021 Apr 16;11:8364. doi: 10.1038/s41598-021-87678-x (PMC8052368; doi:10.1038/s41598-021-87678-x)
Supplement: Supplementary file 3 — Supplementary Information 3. [file 41598_2021_87678_MOESM3_ESM.docx]

**Supplementary material**

**Altered gut microbiome and autism like behavior are associated with parental high salt diet in male mice**

Kazi Farhana Afroz^1^, Noah Reyes^1^, Kobe Young^1^, Kajal Parikh^1, 2^, Varsha Misra^1, 2^ and Karina Alviña* ^1, 3^

^1^Department of Biological Sciences, Texas Tech University, Lubbock, TX 79409.

^2^Honors College, Texas Tech University, Lubbock, TX 79409

^3^ Department of Neuroscience, University of Florida, Gainesville, FL 32610

* Corresponding Author

Correspondence:

Karina Alviña, PhD

1395 Center Drive, Room D5-33D

Gainesville, FL 32610-0244

Office (352) 273 - 8855

[kalvina@ufl.edu](mailto:kalvina@ufl.edu)

**Supplementary figure 1:**

**Male offspring from HSD-fed parents weigh significantly more throughout development**

Body weight of the offspring generation was measured from weaning to adulthood (4 to 8 weeks of age). (a) at 4 weeks old the body weight of both male and female offspring from HSD-fed parents was significantly higher (n=30 in all groups). (b) At 8 weeks old only the male offspring maintained higher body weight (n=16 in all groups). Data are described as mean ± SEM. *p < 0.05, **p < 0.01, ***p <0.001, one-way ANOVA and Tukey’s test.

**Supplementary figure 2:**

**Offspring from HSD-fed parents show no difference in avoidance, rearing or grooming behavior.**

Supplementary Figure 2a shows that the percentage of time spent in the center for male offspring from control-fed parents was 50.81±2.01% vs 57.15±3.02% in males from HSD-fed parents (n=13 from control-fed parents, n=14 from HSD-fed parents; p=0.96, q-Value=0.69). Females showed similar results, with no differences between offspring from parents fed with different diets. The time in the center for females from control-fed dams was 55.56±4.56% (n=12) whereas the center time for females from HSD-fed dams was 48.03±3.12% (n=13; F(3,48)=1.54, Prob>F 0.22, p=0.39, q-Value=2.24; One-way ANOVA, Tukey post hoc test).

From the OFT we also quantified rearing events and did not find significant differences between groups (Supplementary Figure 2b). On average males from control-fed dams reared 11.36±1.48 times (n=11 mice), compared to 8.58±1.17 events quantified for males from HSD-fed dams (n=12 mice; F(3,44)=1.48, Prob>F 0.23; p=0.43, q-Value=2.14; One-way ANOVA, Tukey post hoc test). In females from control-fed dams the number of rearing events reached 8.5±1.27 (n=12 mice) while in females from HSD-fed dams the number was 7.76±1.15 (n=13 mice; p=0.97, q-Value=0.59). Similarly, during the OFT we measured grooming behavior. Supplementary Figure 2c shows that no significant difference was observed between the total grooming time in offspring from control or HSD-fed parents (average grooming time for males from control-fed dams = 29.16±4.48 s, n=13; for males from HSD-fed dams = 26.26±3.23s, n=14; p=0.96, q value=0.70; for females from control-fed dams = 24.31±3.07s, n=12; for females from HSD-fed dams = 29.48±5.50 s, n=13; F(3,48)=0.33, Prob>F 0.80; p=0.83, q-Value=1.21; One-way ANOVA, Tukey post hoc test). Data are described as mean ± SEM. *p < 0.05, **p < 0.01, ***p <0.001, one-way ANOVA and Tukey’s test.
